# Role of cuticular genes in the insect antimicrobial immune response

**DOI:** 10.3389/fcimb.2024.1456075

**Published:** 2024-07-23

**Authors:** Sreeradha Mallick, Ioannis Eleftherianos

**Affiliations:** Infection and Innate Immunity Lab, Department of Biological Sciences, The George Washington University, Washington, DC, United States

**Keywords:** insect immunity, antimicrobial response, host defense, insect cuticle, cuticular genes

## Abstract

Insects are established models for understanding host-pathogen interactions and innate immune mechanisms. The innate immune system in insects is highly efficient in recognizing and opposing pathogens that cause detrimental effects during infection. The cuticular layer which covers the superficial layer of the insect body participates in host defense and wound healing by inducing innate immune responses. Previous studies have started to address the involvement of cuticular genes in conferring resistance to insect pathogens, particularly those that infect by disrupting the insect cuticle. For example, the cuticular gene *Transglutaminase (TG)* in *Drosophila melanogaster* plays a structural role in cuticle formation and blood coagulation and also possesses immune properties against pathogenic infection. However, more information is becoming available about the immune function of other cuticular gene families in insects. In this review, we aim to highlight the recent advances in insect cuticular immunity and address the necessity of pursuing further research to fill the existing gaps in this important field of insect immunology. This information will lead to novel strategies for the efficient management of agricultural insect pests and vectors of plant and human disease.

## Introduction

The innate immune system in invertebrates is considered evolutionarily primitive. Invertebrates and vertebrates share the characteristics of innate immunity, especially the similarities between the insect immune response and the vertebrate innate immune system (Vilmos and Kurucz, 1998). The immune system comprises specialized organs, cell types, and defense mechanisms to restrict pathogen attack. As the first line of defense, the innate immune system detects external threats and regulates responses to eliminate the disease-causing pathogen efficiently (Yu et al., 2022). If the pathogen crosses the exterior physical barrier, the second line of defense involves proteolytic pathways within the body fluid and cellular responses operated by immune cells. A third line of defense includes the production of antimicrobial peptides and effector molecules that actively combat pathogens following the activation of the immune system. These three lines of defense form the main components of the innate immune system and they are phylogenetically conserved across multicellular organisms including mammals and insects.

The outermost lining of the insect body, the epidermis, consists of the exoskeleton which contains layers of cuticle. The cuticular layers are subdivided into the epicuticle which lies in the outermost layer of the cuticle, followed by the exocuticle, and finally, the endocuticle which is the innermost layer. Both the endocuticle and the exocuticle are made up of chitin polysaccharides along with cuticular proteins and therefore they are relatively thick (Ali and Takaki, 2020). In most cases, the overall structure of the insect cuticle is well preserved among different species and is composed of multiple layers with marked components and characteristic features. A wax film and a layer of cement usually protect the epicuticle. The procuticle, which lies beneath the epicuticle, is responsible for most of the cuticular mass. Finally, there is an epidermal cell layer that is present at the cuticular base and controls the secretion of various cuticular elements (Balabanidou et al., 2018).

As the outermost structural tissue of the insect, the cuticle is involved in a vast range of physiological processes that include prevention against desiccation, locomotory functions, detection of environmental stimuli, and mechanical support. The cuticle is the first and primary protector of the insect against extrinsic threats posed by microbial pathogens Also, the cuticle maintains the shape of the insect body and retains water in the organism. Alteration in the amount and composition of cuticular lipids leads to water loss in the insect body. Chemically, the cuticle is made up of proteins, lipids, and chitin (Guo et al., 2022). Based on their amino acid composition, insect cuticular proteins are categorized into several families, such as Cuticular Protein Family (CPF), Cuticular Proteins with Rebers and Riddiford (R&R) Consensus (CPR), CPFL (larva), Cuticular Protein of Low Complexity with conserved Glycine residues (CPLCG), Tweedle (TWDL) (Chen et al., 2018).

Using technical advances in shotgun proteomics and mutant analysis, several families of these insect cuticular proteins have been identified and characterized. For example, more than 200 cuticular genes encoding well-defined protein families have been discovered in *Anopheles gambiae* and *Bombyx mori*. However, it is still unknown whether the CPF and CPFL protein families participate in the formation of the epicuticle or the exocuticle since they are unable to bind to chitin. Besides these known cuticular components, throughout evolution, several other cuticular proteins have evolved in insects (Ali and Takaki, 2020). It is fascinating that more than 1% of the total number of genes in an insect genome include cuticular protein gene families (Pan et al., 2018).

The insect cuticular proteins possess diversified characteristics to perform complex mechanical functions to maintain the cuticle structure. Out of the different insect cuticular protein families characterized so far, the CPR family is the most prevalent, composed of the conserved region Rebers and Riddiford (R&R) consensus. The expanded characteristics of the insect CPR family are also demonstrated by the observation that most of the phytovirus-interacting proteins can be allocated to the three different sub-families of the R&R consensus, and they also participate in defense reactions against circulative and non-circulative plant viruses. It has been shown that CPR1 can bind particles of the rice stipe virus (RSV) in the hemolymph of the vector *Laodelphax striatellus*, also known as the planthopper. This facilitates the movement of the virus towards the salivary gland of the insect vector, thus aiding in virus transmission. Contrary, silencing of the *CPR1* gene in *L. striatellus*, results in a decline in RSV transmission by 57% (Deshoux et al., 2018).

Previously, it was considered that cuticular genes from the same cluster would show similar expression patterns. However, recent research on *Locusta migratoria* has demonstrated that adjacent cuticular genes exhibit differential expression profiles despite belonging to the same cluster. This could be attributed to genomic changes during evolution that led to reorganization of gene regulation (Zhao et al., 2017). Therefore, diverse cuticular genes may be responsible for the regulation of multiple biological functions including immunity. Understanding the immune responses regulated by cuticular genes would be pivotal for bolstering insect defenses against noxious pathogens. The goal of this mini-review is to present recent information on the different cuticular genes that are found in various insect species from four insect orders, namely Diptera, Hymenoptera, Lepidoptera, and Coleoptera. The review also covers findings from previously conducted studies on the immune functional roles of certain cuticular genes in the host insects during infection with diverse pathogens.

## Immune role of cuticular genes in Diptera

Besides its primary role in defense against microbial infections, the cuticular layer also protects insects against adverse environmental conditions and desiccation. Biogenesis of the cuticular layer in *Drosophila melanogaster* occurs during the 16th stage of embryonic development. The cuticular protein-coding genes in *D. melanogaster* belong to the *Nimrod* (*Nim*) gene cluster which is located on the second chromosome. Previous research has established approximately 406 innate immune roles of the proteins encoded by these genes. The different Nim proteins, NimA, NimB1, NimB2, and NimC1 can bind to bacteria and promote phagocytosis. Another Nim protein, NimC4, is involved in the phagocytosis of the glial cells of apoptotic neurons (Cinege et al., 2017).

The Transglutaminases (TG) group of enzymes plays a pivotal role in trapping the invading pathogens and also participates in cuticular morphogenesis and hemolymph clotting. TG represses the Immune deficiency (Imd) signaling pathway in *D. melanogaster* to enhance immune tolerance against beneficial bacteria (Shibata and Kawabata, 2018). Insects possess an open circulatory system, so they are constantly at risk of losing hemolymph (equivalent to blood and lymphatic fluid) when injury occurs. Although the molecular mechanisms might differ, the clotting activity of TG in other animals is very similar to the functioning of the *Drosophila* TG as a coagulation factor (Theopold et al., 2014). Hemolymph coagulation is crucial for maintaining homeostasis and immunity in the larval stages of insects. Among the different clotting factors involved, Fondue is of considerable significance due to its effect on the physical properties of the clot which are regulated by the cuticle. It acts as a substrate for the TG and the *fon* gene is induced following infection. However, Fondue is specific to *Drosophila* and is not conserved in other insect species (Lindgren et al., 2008). Previous research has determined that reduced secretion of TG is associated with increased mortality of fruit flies following injury. Moreover, fruit fly larvae are also vulnerable to infection by entomopathogenic nematodes and their symbiotic bacteria. TG directly targets the microbes when the latter gain access to the hemolymph, which results in the formation of small aggregates and finally segregation by the clot matrix. Previous findings suggest that following phagocytosis of bacteria, sequestration by the clot inhibits the dispersal of the pathogens, and leads to a rapid decrease in bacterial load. Thus, TG-dependent activity in *D. melanogaster* larvae forms a critical defense mechanism against bacterial infection (Wang et al., 2010).

**Figure 1:**
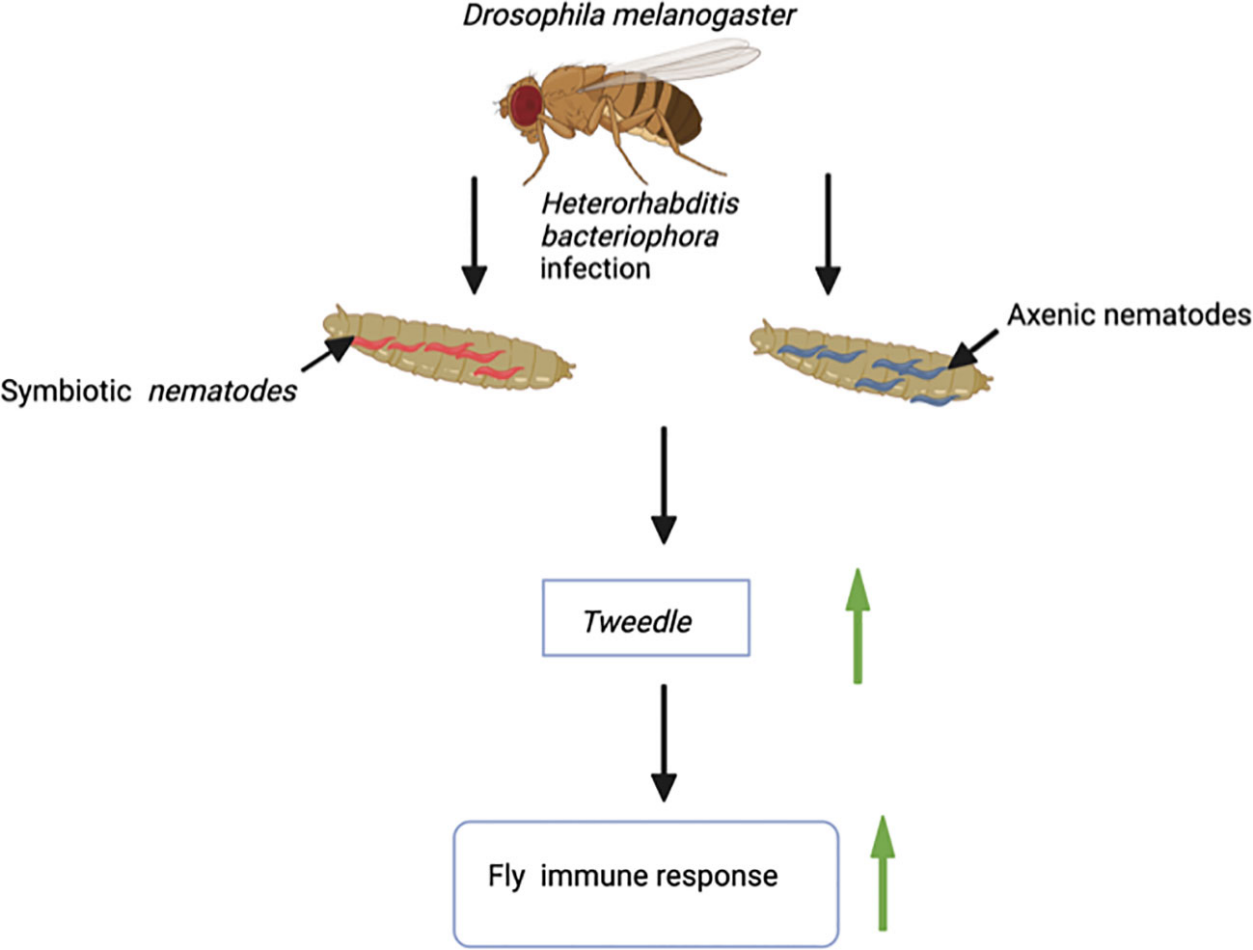
Infection of larvae of the fruit fly *Drosophila melanogaster* with the entomopathogenic nematode *Heterorhabditis bacteriophora* (Symbiotic nematodes, they contain the mutualistic bacteria *Photorhabdus luminescens*; Axenic nematodes, they are devoid of mutualistic bacteria) results in upregulation of the *Tweedle* gene. *Tweedle* gene expression leads to enhanced immune response against entomopathogenic nematode infection.

The *Tweedle* gene family also participates in the cuticular immune response of *D. melanogaster*. A transcriptomic study on *D. melanogaster* adult flies infected with the entomopathogenic nematode *Heterorhabditis bacteriophora* in the absence or presence of its mutualistic bacteria *Photorhabdus luminescens* reported the upregulated expression of several cuticular genes including those from the Tweedle family (**Figure 1**). Notably, *Tweedle* genes are upregulated during the first 30 hours of infection with *H. bacteriophora* axenic nematodes, which are deficient in their *P. luminescens* bacterial symbionts. Following sequence analysis, it is predicted that the *Tweedle* gene family regulates the chitin-binding activity in *D. melanogaster*. Also, the proteins encoded by these genes are important in recognizing invasive parasitic nematodes or opposing nematode migration within the fly tissues (Castillo et al., 2011). Subsequent research has further speculated the role of *Tweedle* in the interaction of insects with parasitic nematodes. An uncharted question in insect immunology involves the characterization of proteins that recognize parasitic nematodes. This raises questions regarding the involvement of *Tweedle* genes in parasitic nematode detection (Eleftherianos and Heryanto, 2021). Therefore, it would be intriguing to investigate whether changes in expression of *Tweedle* genes during entomopathogenic nematode infection activates or circumvents the insect immune response. Moreover, the *Tweedle* gene family, expressed in the tracheae, is involved in warding off *Erwinia carotovora carotovora* 15 (Ecc15) bacteria during oral infection of *D. melanogaster* larvae. The participating *Tweedle* genes perform immune activities by regulating the interaction between the chitinous protective tracheal layer and the bacteria (Castillo et al., 2015). During infection, a new group of genes participating in stress response and oxidoreduction processes are stimulated in the host insect trachea. Among these genes, the expression of 7 members of the chitin-binding proteins in the Tweedle family is downregulated in the trachea. This may be accompanied by a potential conformational change in the thin cuticular layer that guards the trachea. Hence, the Ecc15 bacteria instigate alteration in the *D. melanogaster* larval trachea physiology, events that lead to the activation of immune and stress responses against the bacterial challenge (Gendrin et al., 2013). Moreover, results from sequence analysis have demonstrated that the chitin-binding activity expressed by proteins encoded by *Tweedle* genes makes them excellent targets for understanding host immune responses when fruit flies are infected by parasitic nematodes (Castillo et al., 2015). Research on the *Transglutaminase* and *Tweedle* cuticular gene families will revamp interest in investigating the immune roles of cuticular gene families in Dipteran.

## Immune role of cuticular genes in Hymenoptera

Cuticular genes have an immune role in eusocial insects, like the honey bee *Apis mellifera*. Economically significant honey bee populations are highly susceptible to microbial infections. Honey bees distribute their functional responsibilities among the different members of their colony. While the young workers take care of the offspring, the older members safeguard the nest to protect it from attacks from external intruders. However, variations in functional roles based on the different age groups of honey bees result in differences in their immune response capacity (Luz et al., 2022).

Gene expression during stress in honey bees is affected by the developmental stage. The adult honey bees express enhanced levels of immune-associated factors like phenoloxidase, carboxylesterase, and peptidoglycan recognition protein-S2, during an aseptic and septic injury. These immune signaling molecules boost the immune response of adult honey bees upon injury. However, the expression of these immune components remains unaffected during the early developmental stages. Hence, honey bee larvae lack these immune signaling molecules to for protection against pathogen attacks (Koleoglu et al., 2017).

**Figure 2:**
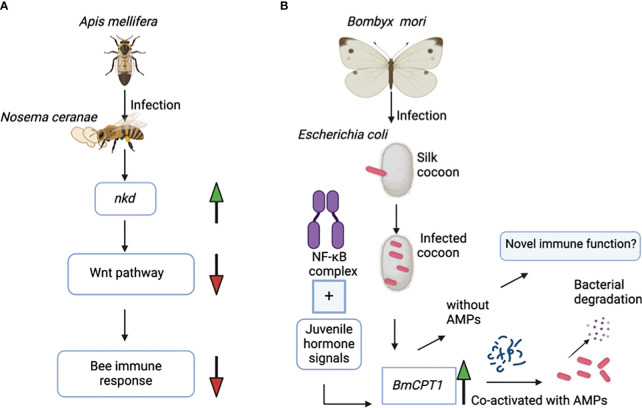
**(A)** Infection of the honey bee *Apis mellifera* with the microsporidian *Nosema ceranae* results in upregulation of the *nkd* gene. Overexpression of *nkd* downregulates the activity of the Wnt pathway, which in turn suppresses the honey bee’s immune response against this parasite. **(B)** The NF-κB immune signaling pathway and the juvenile hormone signals co-regulate the *BmCPT1* gene in the domestic silk moth *Bombyx mori.* Besides being a structural component in the cuticular tissue*, BmCPT1* generates a pattern recognition response in the silk cocoon against pathogenic *Escherichia coli*. *BmCPT1* is co-activated with antimicrobial peptides and results in bacterial degradation.

Interestingly, there is a unique example of how cuticular genes can exert adverse regulatory effects on the immune response of honey bee colonies. The naked cuticle gene (*nkd*), which is also found in *Drosophila* species, negatively regulates the immune system in *A. mellifera*. Exposure of honey bees to the microsporidian parasite *Nosema cerenae* leads to higher expression of the *nkd* gene in the infected bees at 6 and 18 days after infection compared to the uninfected controls. Expression of the *nkd* gene, which functionally opposes the Wingless-related integration site (Wnt) innate immune signaling pathway, leads to downregulation of the host immune response (**Figure 2A**). The Wnt pathway is an evolutionarily conserved pathway that regulates immune responses in both insects and mammals. However, based on previous research, silencing the *nkd* gene in honey bees boosts the immune gene expression, which results in decreased parasite load and therefore enhanced insect survival (Li et al., 2016). The immune role of the *nkd* gene in protection against *N. cerenae* microsporidia triggers interest in investing further efforts to identify the molecular basis of the anti-parasitic immune function of more families of cuticular genes in other hymenopterans.

## Immune role of cuticular proteins in Lepidoptera

The cuticular protein BmCPT1 was first identified in the silkworm *B. mori and* contains a “Tweedle motif”. Overall, three homologs of the *BmCPT1* gene are found in the *B. mori* genome. The NF-κB and the juvenile hormone signals together co-regulate the *BmCPT1* gene in the silkworm (**Figure 2B**) (Futahashi et al., 2008). Silkworms are highly susceptible to pathogen attacks, especially bacterial and viral pathogens. Considering its contribution to the economic importance of the sericulture industry, the silkworm is a suitable natural insect host for studying the regulatory alterations in immune gene expression during pathogen exposure (Xia et al., 2024).

Although bacterial pathogens are a frequent threat to *B. mori*, the mechanism of Gram-negative bacterial detection in the silkworm is relatively unknown. Previous findings suggest that the recognition pattern of Gram-negative bacteria is not conserved between *B. mori* and *D. melanogaster*. However, it has been demonstrated that BmCPT1 is co-activated with a group of antimicrobial peptides to combat Gram-negative bacterial infection (Liang et al., 2015). Antimicrobial peptides form an integral part of innate immunity in insects. Antimicrobial peptides are a group of small peptides, which can be classified based on their amino acid composition, structural features, and functional roles. They are effective against a wide range of microbial pathogens and contribute to disease resistance (Huan et al., 2020). Research in mammalian models has revealed the significant role of antimicrobial peptides in regulating inflammatory responses such as wound healing, psoriasis, and contact dermatitis. They form a specialized barrier in the cuticular skin layer of the mammalian host and also direct innate immune responses (Gallo and Huttner, 1998). Hence, it would be particular interesting to explore the involvement of antimicrobial peptide encoding genes in the cuticular immune response of insects in correlation with the activity of microbial virulence factors.

The antimicrobial peptides provide effective resistance against pathogen invasion and form the backbone of the epithelial barrier. Conserved across invertebrates and vertebrates, previous research has established that the antimicrobial peptide genes operate jointly to act against the invading pathogen and thus strengthen the epithelial innate immunity (Johnstone and Herzberg, 2022). Six different families of antimicrobial peptides have been identified in *B. mori*, which include the following: Gloverin. Cecropin, Defensin, Moricin, Attacin, and Levocin (Makwana et al., 2023). The Toll and the Imd pathways are two key immune signaling cascades that the silkworm activates to combat intruding pathogens. Activation of these pathways regulates the expression of antimicrobial peptide-coding genes (Yang et al., 2018). During microbial infection, the antimicrobial peptides are produced and readily secreted from the fat body into the hemolymph to fight off the pathogens by degrading their cell membrane. Silkworm antimicrobial peptide genes *gloverin*, *BmGlv1*, *BmGlv2, BmGlv3*, and *BmGlv4* are highly expressed in the fat body cells upon *E. coli* infection. Although *BmGlv1* is the primitive member, it evolved and has led to the generation of *BmGlv2, BmGlv3*, and *BmGlv4* genes during duplication in the embryonic stage of the silkworms. This suggests that the evolved genes are expressed in the embryonic stage and also have a novel function (Nesa et al., 2020). Because the cuticle constitutes the epithelial layer of the insect body, future research could explore the possibility of functional cooperation between the antimicrobial peptide genes and the cuticular genes in lepidopterans.

The identification of *BmCPT1* in the cuticular tissues and its subsequent binding to chitin has led to the speculation of its function as a cuticular structural component. However, *BmCPT1* is expressed in both hemocytes and the fat body of the silkworm instead of the cuticle following injection of *E. coli* lipopolysaccharide and peptidoglycan. This observation led to the hypothesis of differential gene regulation of *BmCPT1* in multiple *B. mori* tissues. The current understanding of the function of *BmCPT1* is that it is a structural component of the cuticle and has a morphogenetic role in the absence of pathogen attack. However, *BmCPT1* can also generate a pattern recognition response against Gram-negative bacterial infection. Previous studies in *D. melanogaster* have proposed that the *B. mori* Relish genes, *BmRelish1* and *BmRelish2*, participate in host defense against Gram-negative bacteria. Research with transgenic silkworms has confirmed that overexpression of the *BmCPT1* gene results in transcriptional activation of the Gloverin genes, *BmGlv1 and BmGlv4*, in *B. mori*. This indicates a distinct role of the *BmCPT1* gene in activating and regulating innate immune responses in silkworms (Liang et al., 2015).

## Immune role of cuticular genes in Coleoptera

Entomopathogenic fungi commonly invade insects by penetrating through the cuticular layer. However, the cuticular integument can induce effective anti-fungal responses. The cuticular layer actively guards the host insect by prohibiting the adhesion and germination of fungal spores. The outermost protective cuticular layer, the epicuticle, is the most effective in guarding the insect against fungal pathogens. The cuticle of the insect host also participates in molting, especially by rapid ecdysis, which forms an avoidance mechanism to prevent fungal pathogen invasion (Ma et al., 2024). The presence or absence of certain components of the epicuticle, like phenolic compounds, lipids, and proteins, influence the fungal growth (Włóka et al., 2022). Entomopathogenic fungi target the cuticular layer as a point of entry to the insect hemocoel. Following the introduction of fungal disseminative structures, like conidia, into the insect cuticle, the pathogen establishes a successful infection. Insect cuticles, particularly epicuticles, are highly rich in hydrophobic hydrocarbons and function as a robust barrier system against invading fungal pathogens. Members of the hydrocarbon family like aliphatic and methyl-branched alkanes, serve as growth medium for the fungus *Beauveria bassiana*, which utilizes the hydrocarbons on the insect cuticle and degrades them into simpler organic molecules, like fatty acids and phospholipids. Cuticular components like lipids and waxes that confer resistance to microbial infections vary across the different life stages or instars of the host insects, which forces the pathogenic fungi to adopt alternative approaches to invade the insect cuticle (Ortiz-Urquiza and Keyhani, 2013).

Recent work in the red flour beetle *Tribolium castaneum* has revealed that pupal survival is substantially elevated when the pathogenic fungus *B. bassiana* penetrates the insect cuticle, while extensive mortality occurs when the beetles are microinjected within the hemocoel with a solution containing a small number of yeast-like hyphal cells. This observation has demonstrated the effectiveness of the cuticular layer in providing sufficient protection against entomopathogenic fungal invasion. Furthermore, knocking down the cuticular gene *chitin synthase 1* in the pupal stage leads to increased sensitivity to transcutaneous infections by the pathogenic fungi *B. bassiana* and *Metarhizium anisopliae* in adult beetles (Sawada et al., 2020).

*Tribolium castaneum* is a widespread invasive insect pest of stored agricultural products like nuts, spices, legumes, and flour (Johnson, 2013). Besides being an insect pest, the red flour beetle is a well-established insect model for analyzing the immune system in Coleoptera due to its genetic tractability. These beetles possess protein-coded genes with conserved sequences that regulate the Toll immune signaling pathway (Altincicek et al., 2008). Previous research involving adult-specific cuticular genes, *CPR* such as *CPR4, CPR18, and CPR 27*, has established that these genes confer anti-fungal immune properties to the cuticular layer of adult beetles. The transcript level of the three *CPR* genes does not increase significantly and their expression decreases during the pupal stage. Although the mRNA levels of the *CPR18* gene are elevated in second-day pupae, this gene expression pattern is observed only in the latter half of the pupal stage. Despite showing a similar trend in expression pattern with the *CPR18* gene in stage 2 pupae, *CPR4* is not substantially expressed in the remaining pupal stages. The *CPR4* gene was most effective in forming the pore canal during the adult stages. The *CPR*2*7* gene, however, unlike the *CPR4*, is not expressed in adult beetles but is mostly induced in the middle and final pupal stages of *T. castaneum* (Sirasoonthorn et al., 2021). However, the abundance of the *CPR* gene family might vary among insects, and *CPR* genes possess characteristic motifs that are conserved in insect vectors like *Anopheles gambiae* (Wang et al., 2019). Expanding this research will provide critical information for devising novel strategies to combat coleopteran insect pests with entomopathogens that target the cuticle.

## Conclusions and perspectives

Pathogens have evolved strategies to overcome the insect immune response. Currently, there is a significant lack of information on the role of cuticular genes in shaping the host insect defense against pathogen infections. Most studies have mainly focused on the involvement of a selected number of insect cuticular genes in the regulation of developmental processes. However, accumulated evidence from recent work has started to document that the cuticular layer is not only a simple physical barrier guarding the entry of pathogens. This new information establishes the insect cuticular layer as a critical regulator of homeostasis and innate immune function. Future studies in various insect species and stages will focus on the dissection of the molecular mechanisms that allow cuticular genes to modify the host immune response and determine whether these mechanisms vary with the type of pathogen. Another line of research could involve the investigation of a potential correlation between the number of pathogens attacking the insect cuticle and the potency of different facets of the insect immune response in the cuticle as well as in other tissues. Although previous work on the Tweedle cuticular gene *BmCPT1* was performed in *B. mori* in the context of *E. coli* infection (Liang et al., 2015), future efforts could concentrate on the identification of the *Tweedle* gene family members which regulate the immune response against pathogen invasion. Also, other cuticular gene families such as *CPF* and *CPLCG*, could be explored to assess the differential expression of their gene members in insects exposed to microbial pathogens and eukaryotic parasites that penetrate the cuticle. A significant aspect of future research would be to perform parallel studies in model insects as well as natural insect hosts to determine the conservation of the immune role of cuticular gene families in adjusting the innate immune recognition, signaling activity, and expression of effector molecules to oppose pathogenic infection. Extensive destruction of economically significant insect populations like the silkworm and the honeybee can only be prevented if there is sufficient understanding of the exact nature and role of cuticular genes during the early stages of infection. The availability of this type of information will contribute towards developing alternative agricultural tactics involving potent entomopathogens for eliminating destructive insect pests and disease vectors. In the long run, understanding the broad immune function of conserved cuticular genes will pave the way for establishing novel approaches for elucidating the cuticular mechanisms of defense in vertebrate animals, including humans, thereby facilitating progress in biomedical research.

## Author contributions

SM: Conceptualization, Writing – review & editing, Validation, Visualization, Writing – original draft. IE: Conceptualization, Writing – review & editing, Funding acquisition, Supervision.
